# Supplementary material for: Anticancer Treatment Goals and Prognostic Misperceptions among Advanced Cancer Outpatients
Source: Int J Environ Res Public Health. 2022 May 21;19(10):6272. doi: 10.3390/ijerph19106272 (PMC9141160; doi:10.3390/ijerph19106272)
Supplement: Supplementary file 1 [file ijerph-19-06272-s001.zip › ijerph-1696823-supplementary.pdf]

# Supplementary Material

Patient ID: __________

Date of collection: __/__/____

## COMMUNICATION ASSESSMENT TOOL - PATIENT FORM

You have just been seen by the doctor and received some news about your health condition.
Please answer the questions below, considering what best describes your conversation with the doctor, checking all the phrases with which you can identify. Please be as honest as possible, and remember that all information is kept confidential.

Patient's name:

Date of birth:

Attending Physician (Name/ID):

### QUESTIONS ABOUT YOUR STATE OF HEALTH AND THE NEWS RECEIVED TODAY

1. in which organ (body part) did your cancer begin?

( ) Breast; ( ) Cervix uteri; ( ) Ovary; ( ) Endometrium - uterus; ( ) Colorectal; ( ) Stomach; ( ) Esophagus; ( ) Liver
( ) Bile ducts; ( ) Pancreas; ( ) Prostate; ( ) Bladder; ( ) Kidney; ( ) Testicle;
( ) Other (specify:_____________________________________________________)

2. Can you tell where your disease (your cancer) is located at the moment?

( ) Locoregional; ( ) Non-regional lymph nodes; ( ) Muscle / Skin / subcutaneous; ( ) Bone(s); ( ) Lung(s); ( ) Liver; ( ) Central Nervous System; ( ) Adrenal; ( ) Spleen; ( ) Peritoneum; ( ) Don't know
( ) Other places (specify: ____________________________________)

3. Talking about cure is always complex, since it involves what you wish (would like) and what you think might actually happen. To answer this question, think only about what you believe can really happen, without taking your wish or faith into consideration.
In your opinion, what is your **chance of a cure** (of being completely free of cancer)?

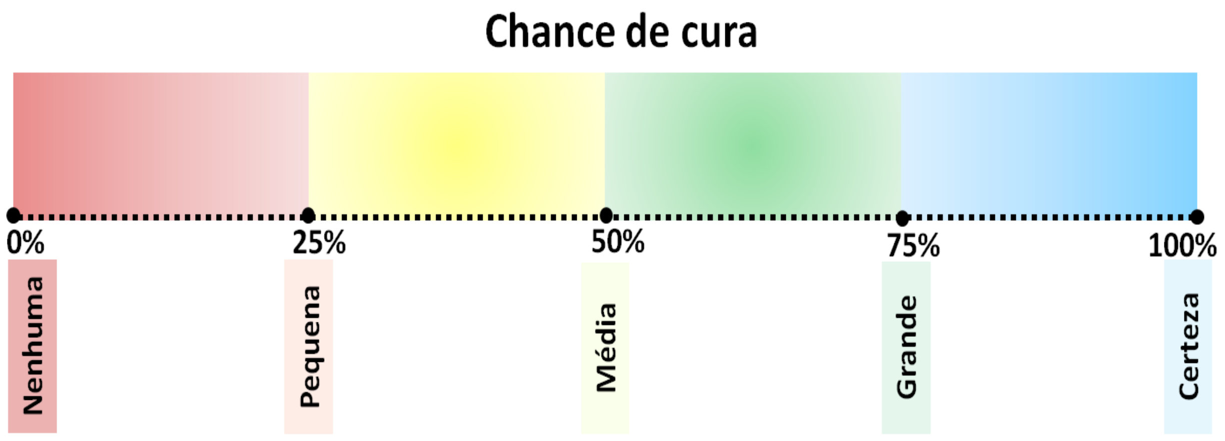


4. What information did the doctor explain to you about your treatment?
(You can check more than one option).

( ) That I need to receive chemotherapy against cancer
( ) That I need to receive other treatment against cancer (specify:____________________________________)
( ) That I am too weak to receive treatment against cancer (chemotherapy, for example)
( ) That there are no medicines against cancer that can help me
( ) That the most important thing now is the control of the symptoms caused by cancer, and that this is done by a team specialized in palliative care.
( ) That I can return to treatment against cancer if I improve my clinical conditions.
( ) Did not explain me anything about my treatment

( ) Other information. (specify:_____________________________________________________)

5. Can you tell me the main treatment goal that the doctor prescribed for you today?
( ) The goal of treatment is to completely CURE my cancer.
( ) The goal of treatment is to INCREASE my life span as much as possible.
( ) The goal of treatment is to ALLEVIATE my symptoms and make me not suffer.
( ) Not applicable (no treatment prescribed).
( ) Other option (specify:_________________)

6. In today's consultation, you helped your doctor decide on the treatment for your disease

| ( ) **YES**<br>*if yes, follow up by answering this column* | ( ) **NO**<br>*if no, follow up by answering this column* |
|---|---|
| Did you really want to have participated in this decision? ( ) Yes ( ) No | Did the doctor decide on his own?<br>( ) Yes ( ) No<br>Did it bother you?<br>( ) Yes ( ) No |
| Did your family also help the doctor to decide?<br>( ) Yes ( ) No<br>Did it bother you?<br>( ) Yes ( ) No | Did your family help the doctor to decide?<br>( ) Yes ( ) No<br>Did it bother you?<br>( ) Yes ( ) No |
| | Would you like to have participated in the treatment decision?<br>( ) Yes ( ) No |

7. Good doctor-patient communication includes the doctor listening carefully to what the patient has to say, letting the patient feel free to ask questions, explaining information about their health clearly. So...
Considering zero as bad and ten as excellent, how would you rate your doctor's behavior today when communicating with you?

| 0 | 1 | 2 | 3 | 4 | 5 | 6 | 7 | 8 | 9 | 10 |
|---|---|---|---|---|---|---|---|---|---|---|

**poor communication** **excellent communication**

## PATIENT PREFERENCES REGARDING COMMUNICATION

People, in general, have different profiles: some like to receive very detailed information about their disease, life expectancy, and treatment; others, although they want to know truthful information, prefer to know only superficially about the situation. There are also those who prefer not to know anything about their disease. Knowing this, what are your preferences regarding...

8. Regarding information about your treatment, how would you prefer the doctor to explain it to you?
( ) I like to know all the details[1]
( ) I like to know only the main information[2]
( ) I don't like to know anything[3]

( ) Did not know how to answer.
( ) Did not want to answer.
( ) Other answer (specify: ______________________)

9. Do you think it is important that your doctor tells you about your prognosis (chances of cure and life span)?
( ) Yes (go to item 10)
( ) No (go to item 11)

( ) Did not know how to answer.
( ) Did not want to answer.
( ) Other answer (specify: ______________________)

| Item 10. Since you think it is important for the doctor to tell you about your prognosis, how would you like to receive the information? |
|---|
| ( ) I think it is important to know as much detail as possible about my prognosis<br>( ) I think it's important to have a general idea, but not too many details.<br><br>*( ) Did not know how to answer.*<br>*( ) Did not want to answer.*<br>( ) Other answer (specify: _______________________) |

| Item 11. You said that you don't think it is important that your doctor tells you about your prognosis (chances of cure, length of life). Why is this? |
|---|
| ( ) I see no reason to know (would not change my life at all)<br>( ) I think it can do me harm<br>( ) I don't believe in the life time estimates made by doctors<br><br>*( ) Did not know how to answer.*<br>*( ) Did not want to answer.*<br>( ) Other answer (specify: _______________________) |

| Item 12. When talking about your prognosis (chances of cure, length of life) or expectations about your treatment, how would you prefer the doctor to explain it to you? |
|---|
| ( ) That he be realistic<br>( ) That he be realistic, but try to keep my hope with positive information<br>( ) That he only worries about keeping my hope with positive information<br><br>( ) Did not know how to answer.<br>( ) Did not want to answer.<br>( ) Other answer (specify: _______________________) |

| **ASSESSMENT OF MEDICAL COMMUNICATION** |
|---|
| 13. Regarding today's appointment, did the doctor talk about your chances of cure and length of life? |
| ( ) Yes (go to item 14)<br>( ) No (go to item 15) |
| 14. Since the doctor told you about your chances of cure and length of life, how would you rate the amount of information he told you? |
| ( ) I would like to have received MORE information;<br>( ) I received enough information;<br>( ) I would like to have received LESS information.<br><br>*( ) Could not answer.*<br>*( ) Did not want to answer.*<br>( ) Other answer (specify: ) |

| 15. Regarding today's appointment, how do you rate the information the doctor gave you about your treatment? |
|---|
| ( ) I would like to have received MORE information;<br>( ) I received enough information;<br>( ) I would like to have received LESS information.<br><br>( ) Could not answer.<br>( ) Did not want to answer.<br>( ) Other answer (specify: ) |

**CARE PREFERENCES**

16. If you could choose, would you prefer...
*choose only one of the options*

( ) A treatment that was designed to prolong his life as much as possible, even if the treatment caused more pain and discomfort
( ) A care plan to relieve pain and discomfort as much as possible, even if it meant not living as long

Advance Care Planning is a process of discussing and recording the person's PREFERENCES for care in the future, when they are no longer able to make decisions on their own. It takes into consideration each person's values and life goals. Advance Care Planning can be discussed at any time by both healthy people and people who are ill.

17. Have you and your doctor discussed, today or during recent visits, any particular wishes you have about the care you would like to receive if you were dying?
(To answer this question, consider not only today's visit, but also recent visits.)

( ) Yes
( ) No

( ) Did not want to answer
( ) Doesn't remember
( ) Other answer (specify: )

18. If not, do you think it is important to talk with your doctor about these issues at some point?

( ) Yes
( ) Maybe
( ) No

( ) Did not want to answer
( ) Other answer (specify: )

19. Have you and any family members ever talked at any time about any particular wishes you have about the care you would like to receive if you were dying?

( ) Yes
( ) No

( ) Did not want to answer
( ) Doesn't remember
( ) Other answer (specify: )

Note: Literally translated from Portuguese (original) into English.

## COMMUNICATION ASSESSMENT TOOL - PHYSICIAN'S FORM

Physician's Full Name:

Age:

Graduation year in medicine:

Function: ( ) Tenured physician; ( ) R3; ( ) R2; ( ) R1

Year of training as oncologist (for tenured):

Gender: ( ) Male ( ) Female

This questionnaire is confidential. Please be as honest as possible when answering it.
Please mark on each question all the statements that you can identify with.

Patient's full name:

1. Did you estimate the patient's prognosis at this visit? (If you have thought about or considered the prognosis in any way, even without calculations or objective tools, mark YES)

( ) No (go to item 4)
( ) Yes (go to item 2)

2. In what way did you estimate the prognosis of the patient you saw?

( ) Only based on my practical experience
( ) Prognostic scales (e.g. PPI, PaP, among others)
( ) Performance status scales
( ) Laboratory exams
( ) HAprog
( ) Other answer (specify: ________________________)

3. Considering this consultation, in what way did the prognostic estimate influence your medical conduct (for example, in the indication or not of chemotherapy, suspension of treatment, hospitalization, referral to palliative care, etc)?

( ) It did not influence at all
( ) Influenced a little
( ) Moderately Influenced
( ) Influenced a lot
( ) Influenced totally

4. In your opinion, what are the chances of cure for the patient:

( ) 0%; ( ) <10%; ( ) 10 a 24%; ( ) 25 to 49%; ( ) 50 to 74%; ( ) 75 to 90%; ( ) more than 90%

5. What is your estimate of survival for the patient in question (remaining life span; in months)?

___________months

6. If your estimate above (in item 8) is < 6 months, could you estimate in days?

___________ days

7. Mark with an X the probabilities of survival (chances of the patient being alive), ranging from 0% to 100%, at the following times:

| Moments | 0% | 10% | 20% | 30% | 40% | 50% | 60% | 70% | 80% | 90% | 100% |
|---|---|---|---|---|---|---|---|---|---|---|---|
| 1 month | | | | | | | | | | | |
| 3 months | | | | | | | | | | | |
| 6 months | | | | | | | | | | | |
| 1 year | | | | | | | | | | | |

8. If the patient in question lived significantly LESS than your clinical estimate of survival, would your therapeutic proposal be different?

( ) Yes, describe: ____________________________________________
( ) No

( ) Not applicable (I did NOT estimate the patient's prognosis in any way)

9. If the patient in question lived significantly longer than your clinical estimate of survival, would your therapeutic proposal be different?

( ) Yes, describe: ___________________________________________________

( ) No

( ) Not applicable (I did NOT estimate the patient's prognosis in any way)

10. Did you tell the patient the whole reality about the disease?

( ) YES, frankly and realistically (go to item 12)

( ) I told almost the whole reality (go to item 12)

( ) I told only a SMALL part of it (go to item 11)

( ) I did NOT tell the reality of the disease (go to item 11)

11. For what reasons did you prefer not to tell the reality of the disease?

Answer:

12. Have you explained to the patient what the main goals of the treatment will be from now on?

( ) Yes, I said it would cure cancer.

( ) Yes, I said it would increase the length of life.

( ) Yes, I said it would improve the quality of life / decrease symptoms.

( ) I did not enter into details about the objectives of treatment.

( ) Not applicable (I did not prescribe antineoplastic treatment)

Note: Literally translated from Portuguese (original) into English.
